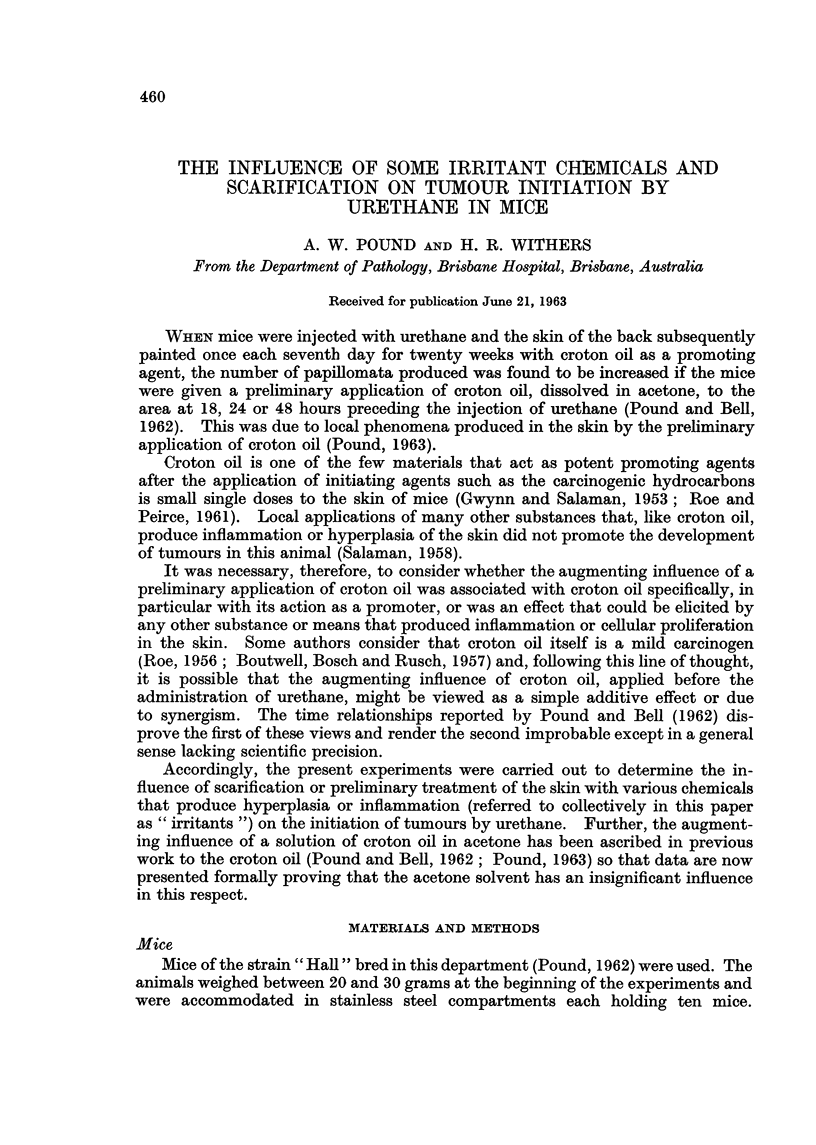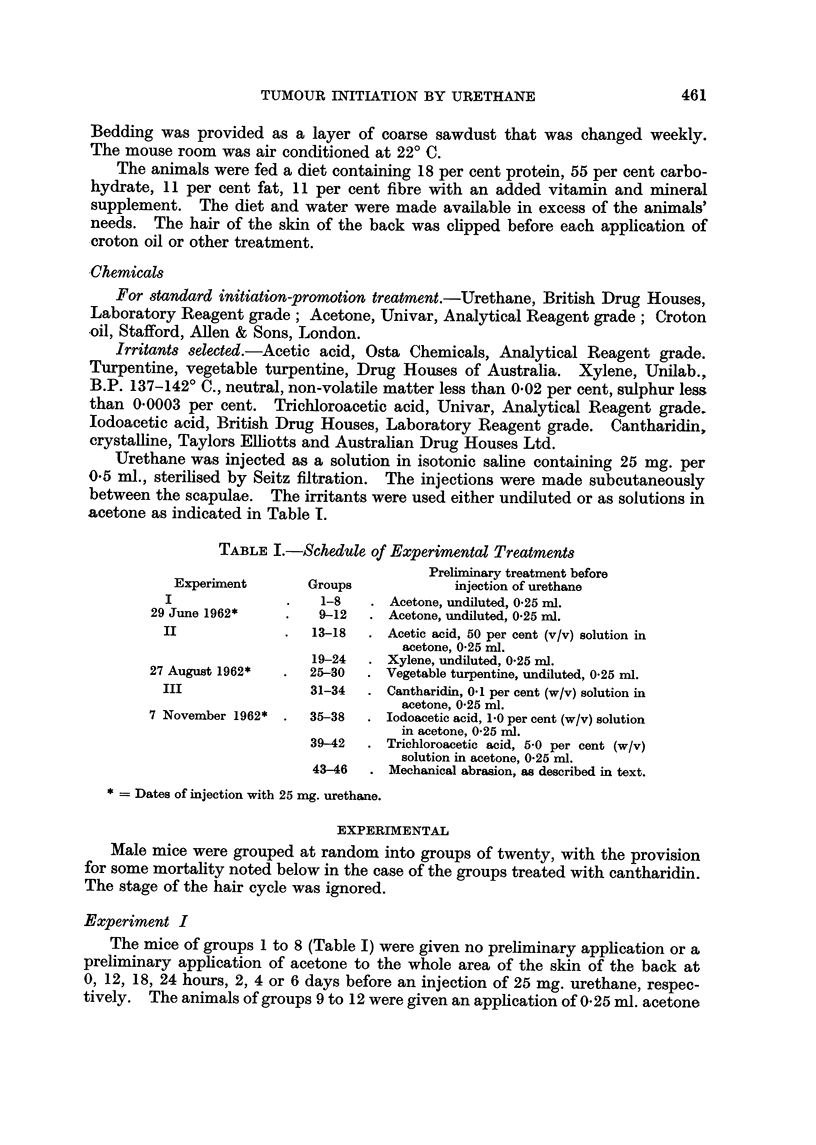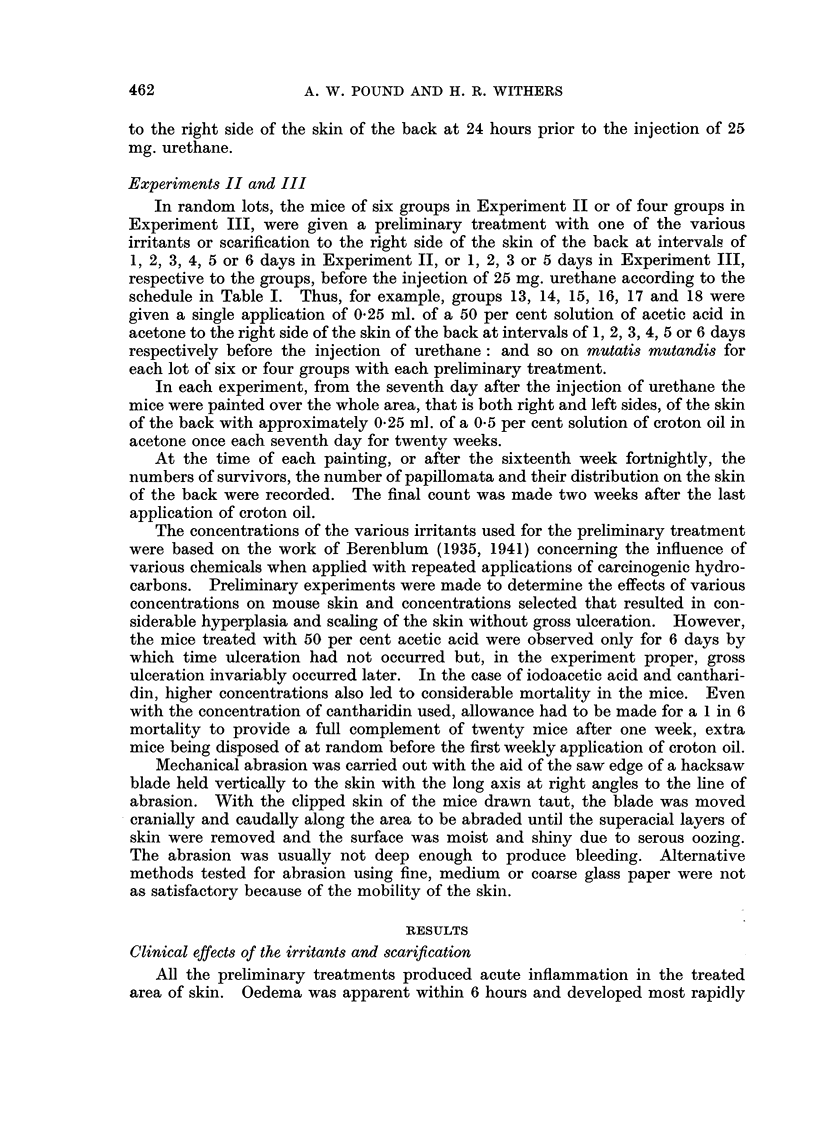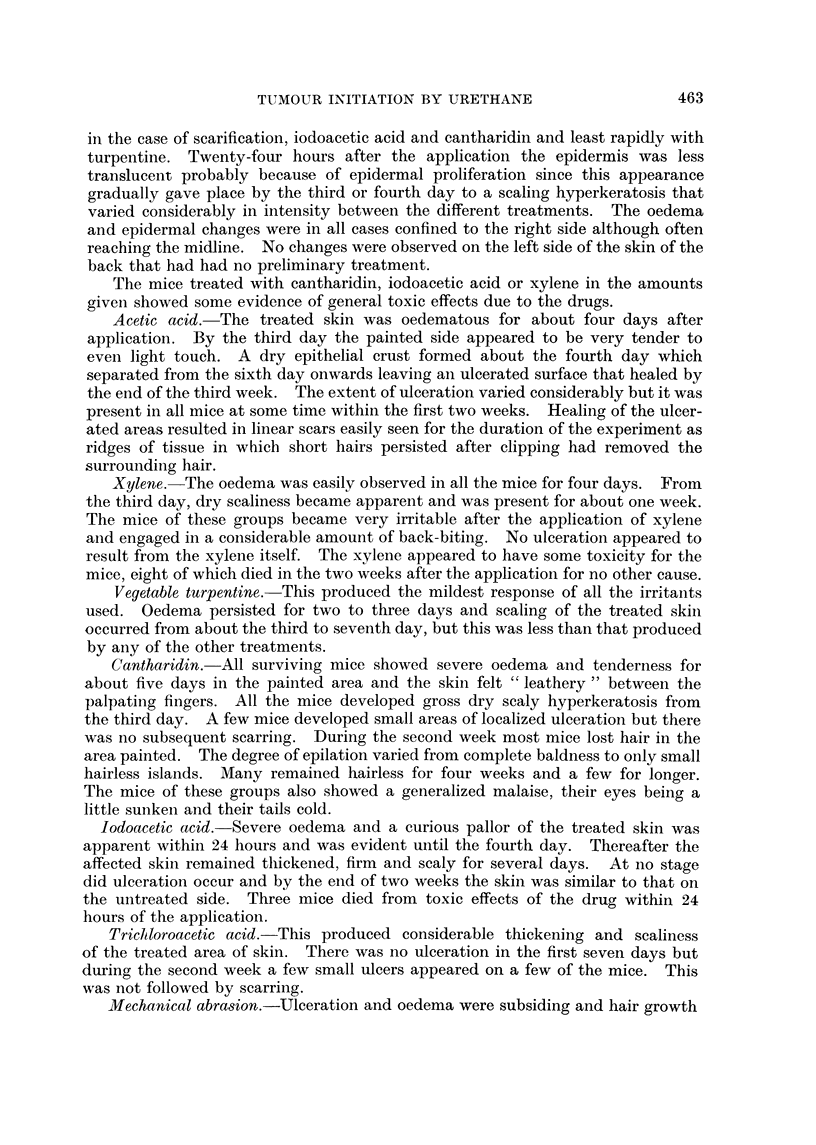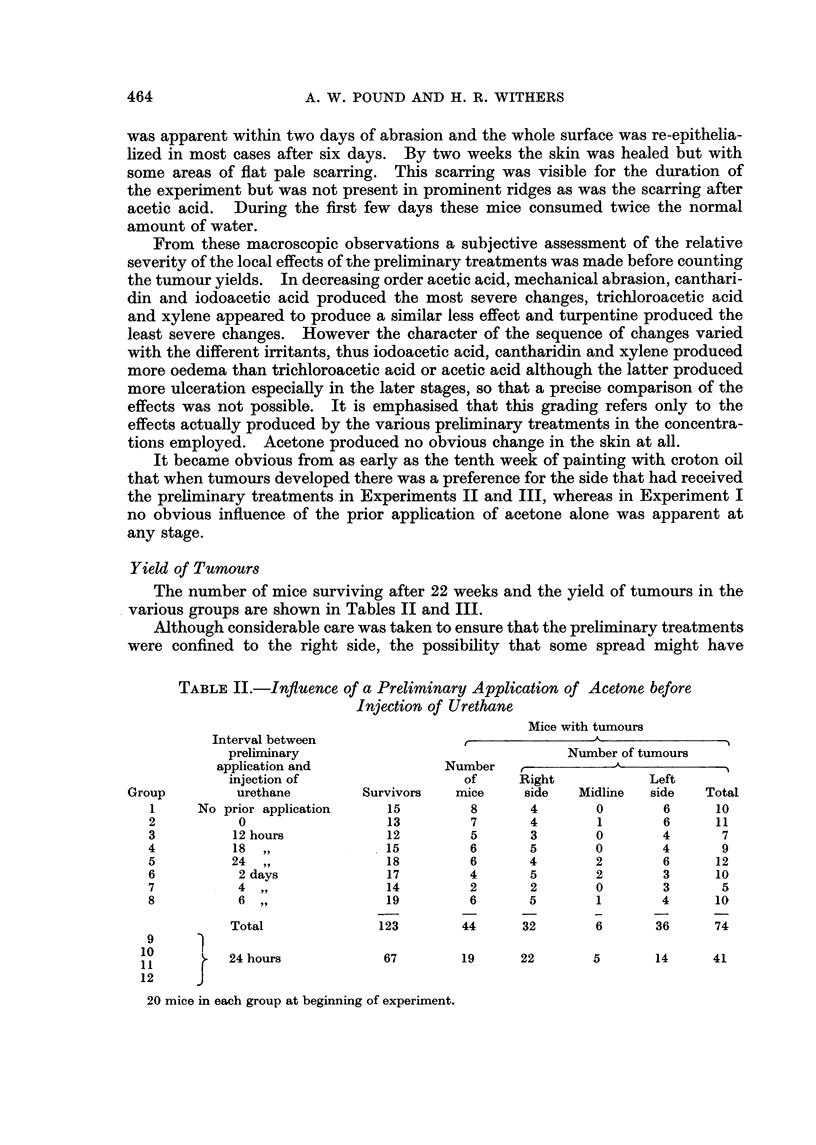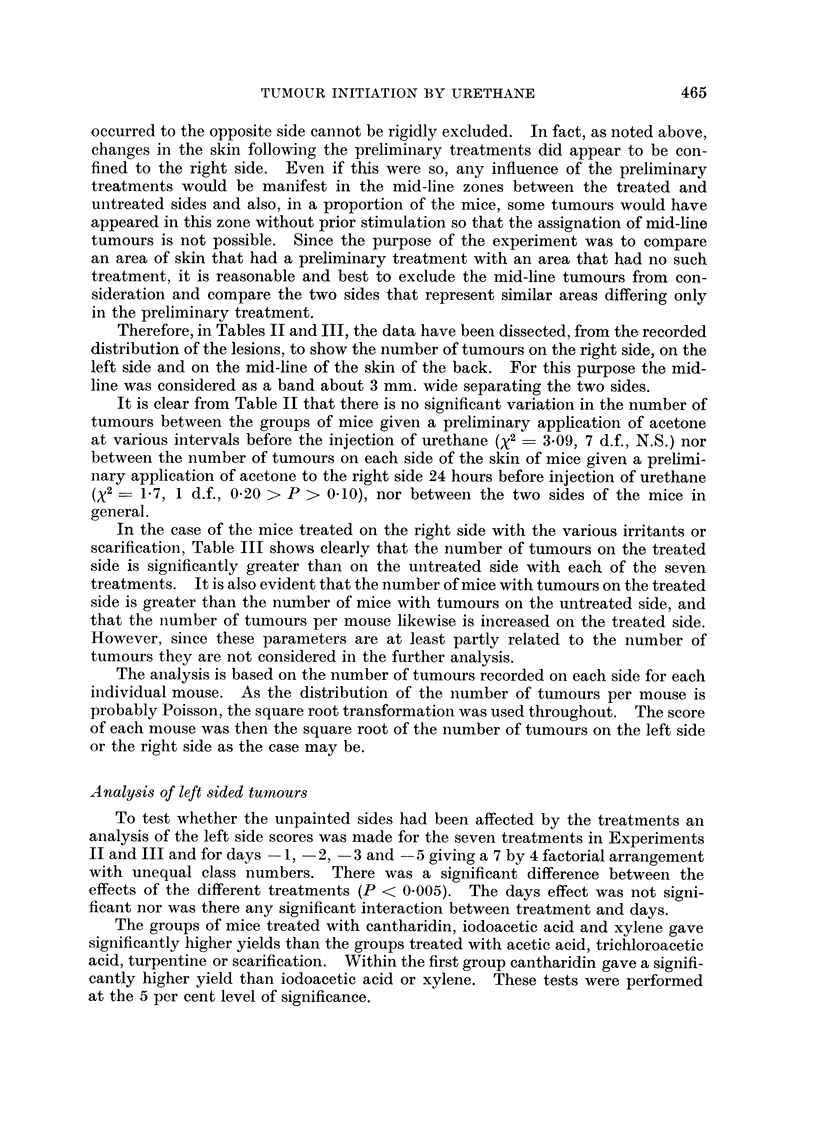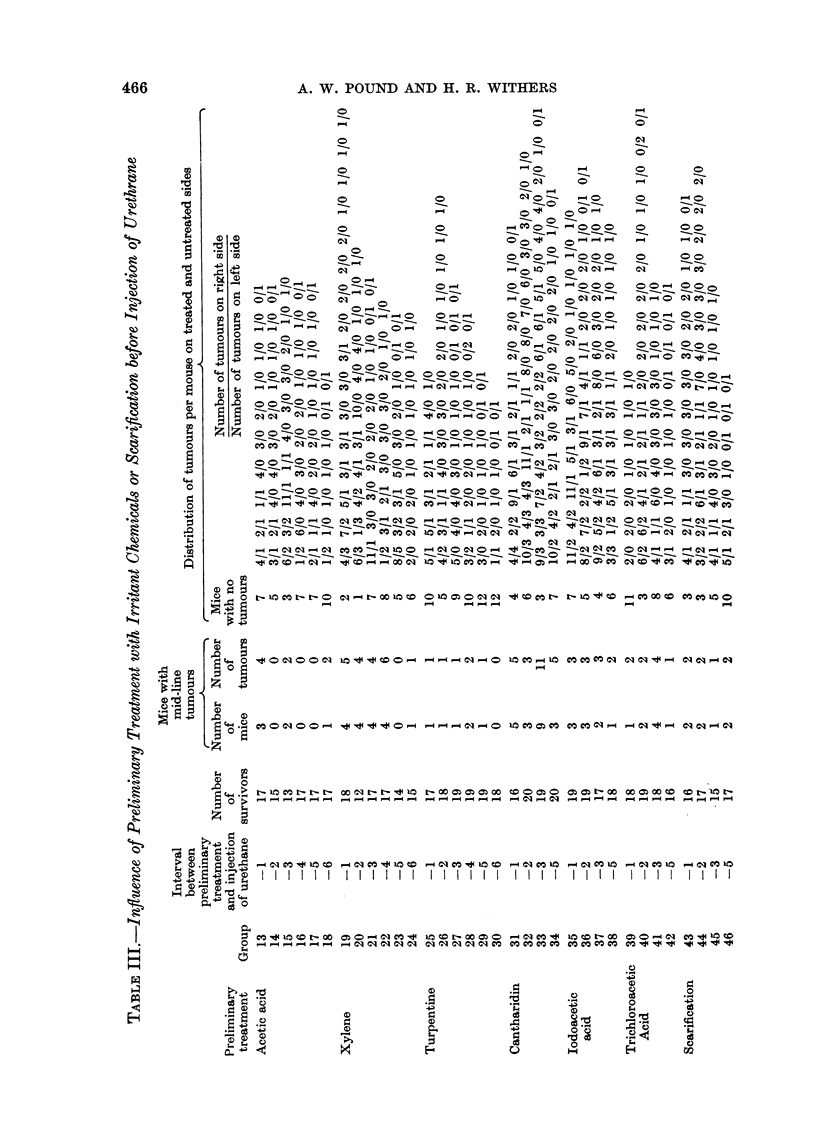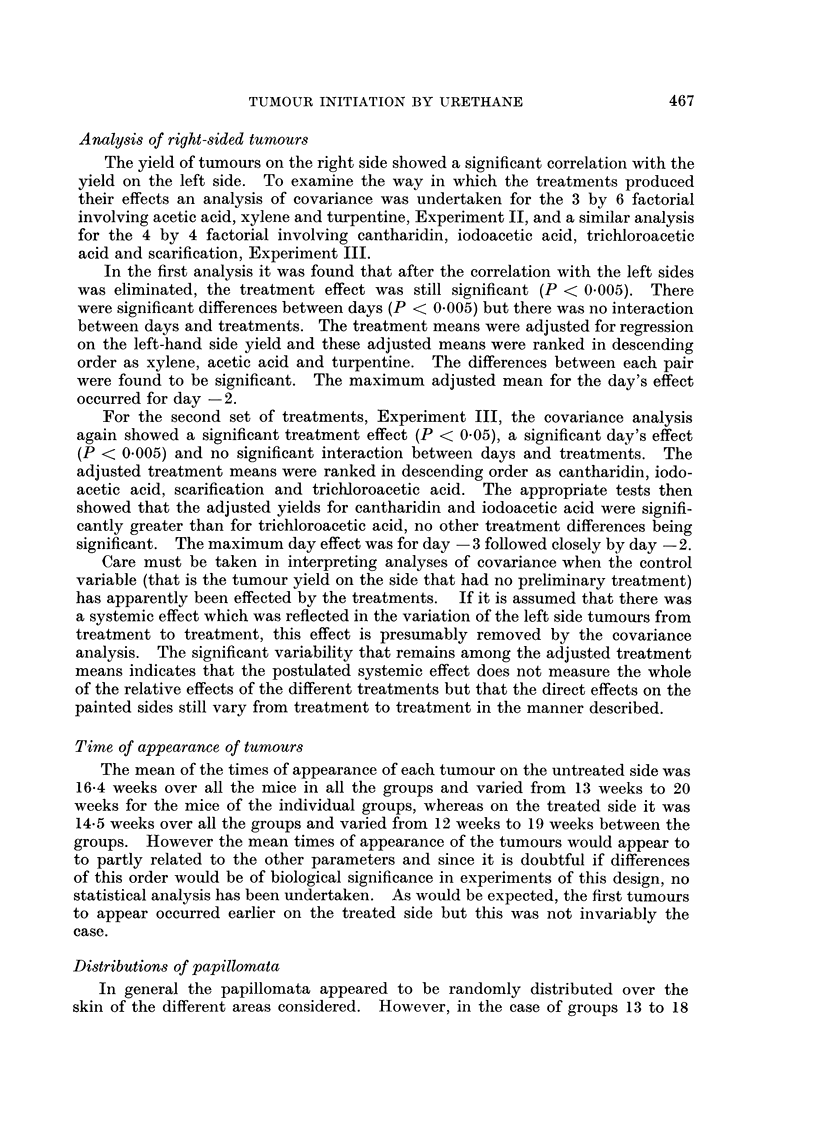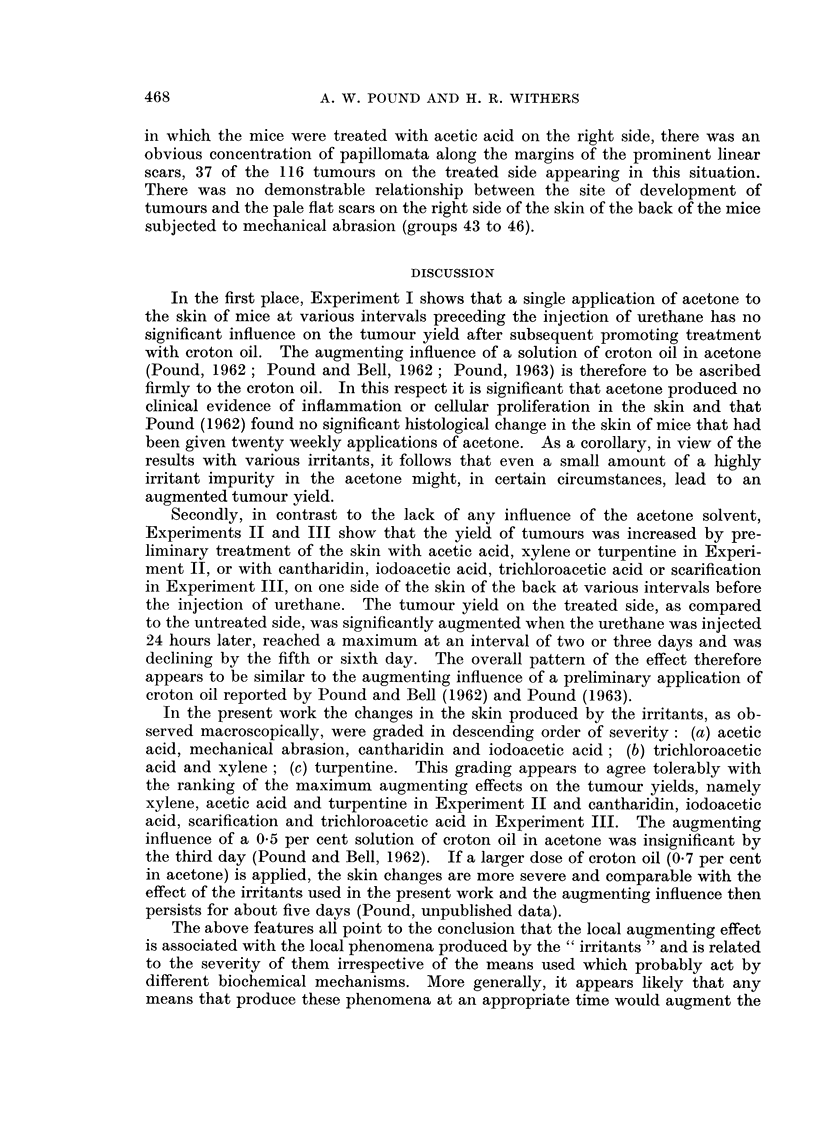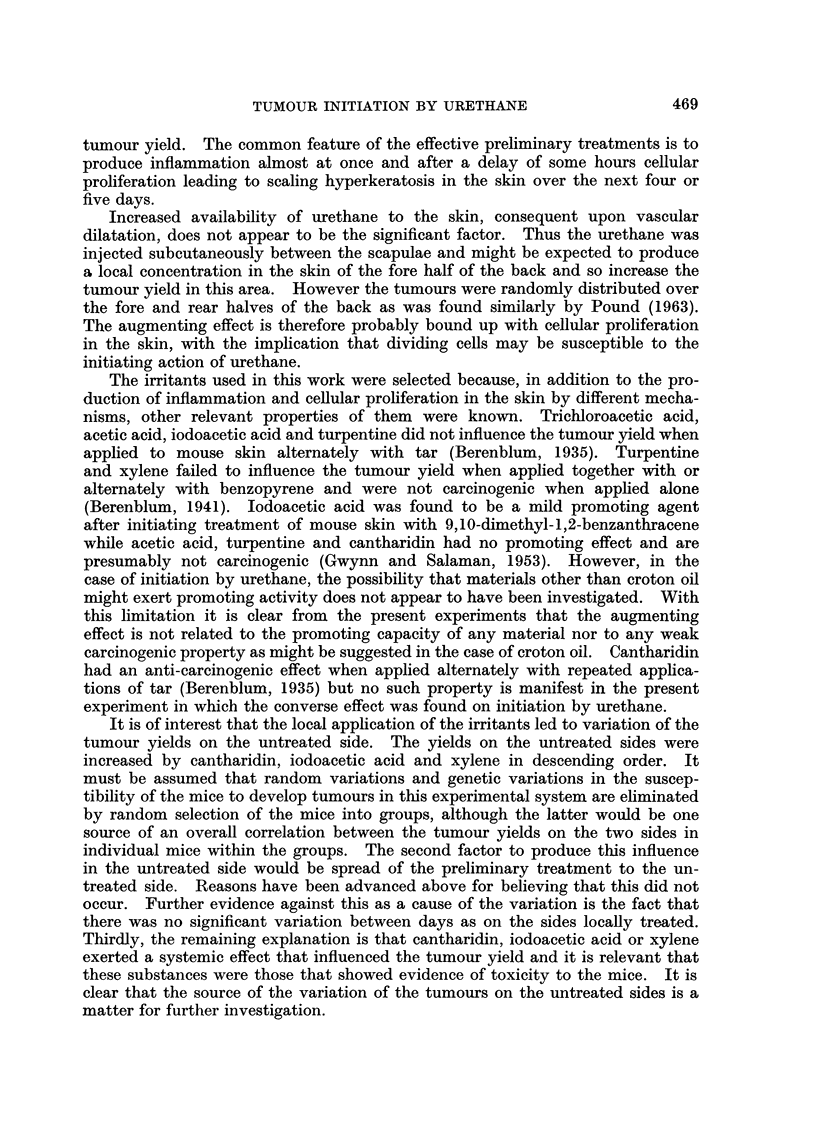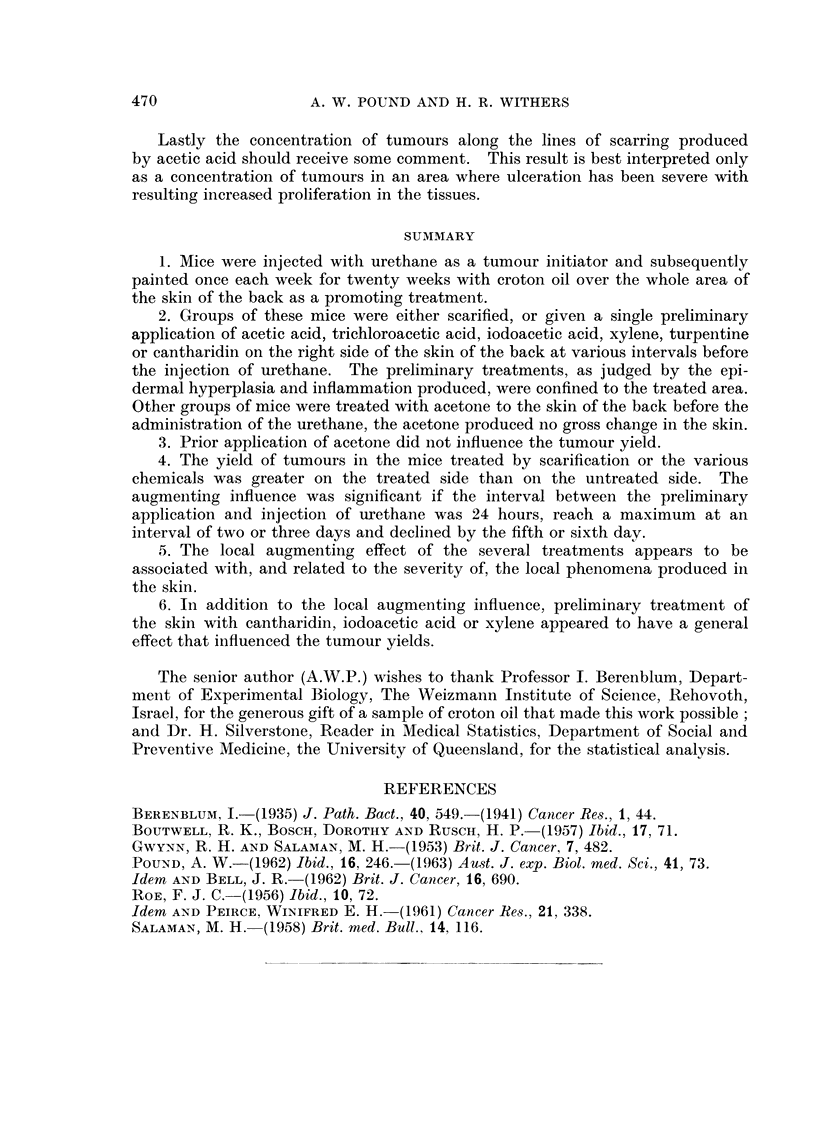# The Influence of Some Irritant Chemicals and Scarification on Tumour Initiation by Urethane in Mice

**DOI:** 10.1038/bjc.1963.61

**Published:** 1963-09

**Authors:** A. W. Pound, H. R. Withers


### THE INFLUENCE OF SOME IRRITANT CHEMICALS AND SCARIFICATION ON TUMOUR INITIATION BY URETHANE IN MICE

A. W. POUND AND H. R. WITHERS

*From the Department of Pathology, Brisbane Hospital, Brisbane, Australia*

Received for publication June 21, 1963

WHEN mice were injected with urethane and the skin of the back subsequently painted once each seventh day for twenty weeks with croton oil as a promoting agent, the number of papillomata produced was found to be increased if the mice were given a preliminary application of croton oil, dissolved in acetone, to the area at 18, 24 or 48 hours preceding the injection of urethane (Pound and Bell, 1962). This was due to local phenomena produced in the skin by the preliminary application of croton oil (Pound, 1963).

Croton oil is one of the few materials that act as potent promoting agents after the application of initiating agents such as the carcinogenic hydrocarbons is small single doses to the skin of mice (Gwynn and Salaman, 1953 ; Roe and Peirce, 1961). Local applications of many other substances that, like croton oil, produce inflammation or hyperplasia of the skin did not promote the development of tumours in this animal (Salaman, 1958).

It was necessary, therefore, to consider whether the augmenting influence of a preliminary application of croton oil was associated with croton oil specifically, in particular with its action as a promoter, or was an effect that could be elicited by any other substance or means that produced inflammation or cellular proliferation in the skin. Some authors consider that croton oil itself is a mild carcinogen (Roe, 1956 ; Boutwell, Bosch and Rusch, 1957) and, following this line of thought, it is possible that the augmenting influence of croton oil, applied before the administration of urethane, might be viewed as a simple additive effect or due to synergism. The time relationships reported by Pound and Bell (1962) disprove the first of these views and render the second improbable except in a general sense lacking scientific precision.

Accordingly, the present experiments were carried out to determine the influence of scarification or preliminary treatment of the skin with various chemicals that produce hyperplasia or inflammation (referred to collectively in this paper as " irritants ") on the initiation of tumours by urethane. Further, the augmenting influence of a solution of croton oil in acetone has been ascribed in previous work to the croton oil (Pound and Bell, 1962 ; Pound, 1963) so that data are now presented formally proving that the acetone solvent has an insignificant influence in this respect.

### MATERIALS AND METHODS

### *Mice*

Mice of the strain "Hall" bred in this department (Pound, 1962) were used. The animals weighed between 20 and 30 grams at the beginning of the experiments and were accommodated in stainless steel compartments each holding ten mice.

Bedding was provided as a layer of coarse sawdust that was changed weekly. The mouse room was air conditioned at 22° C.

The animals were fed a diet containing 18 per cent protein, 55 per cent carbohydrate, 11 per cent fat, 11 per cent fibre with an added vitamin and mineral supplement. The diet and water were made available in excess of the animals' needs. The hair of the skin of the back was clipped before each application of croton oil or other treatment.

*Chemicals*

*For standard initiation-promotion treatment.*—Urethane, British Drug Houses, Laboratory Reagent grade ; Acetone, Univar, Analytical Reagent grade ; Croton oil, Stafford, Allen & Sons, London.

*Irritants selected.*—Acetic acid, Osta Chemicals, Analytical Reagent grade. Turpentine, vegetable turpentine, Drug Houses of Australia. Xylene, Unilab., B.P. 137–142° C., neutral, non-volatile matter less than 0·02 per cent, sulphur less than 0·0003 per cent. Trichloroacetic acid, Univar, Analytical Reagent grade. Iodoacetic acid, British Drug Houses, Laboratory Reagent grade. Cantharidin, crystalline, Taylors Elliotts and Australian Drug Houses Ltd.

Urethane was injected as a solution in isotonic saline containing 25 mg. per 0·5 ml., sterilised by Seitz filtration. The injections were made subcutaneously between the scapulae. The irritants were used either undiluted or as solutions in acetone as indicated in Table I.

TABLE I.—*Schedule of Experimental Treatments*

| Experiment | Groups | Preliminary treatment before injection of urethane |
|---|---|---|
| I | 1–8 | Acetone, undiluted, 0·25 ml. |
| 29 June 1962* | 9–12 | Acetone, undiluted, 0·25 ml. |
| II | 13–18 | Acetic acid, 50 per cent (v/v) solution in acetone, 0·25 ml. |
| | 19–24 | Xylene, undiluted, 0·25 ml. |
| 27 August 1962* | 25–30 | Vegetable turpentine, undiluted, 0·25 ml. |
| III | 31–34 | Cantharidin, 0·1 per cent (w/v) solution in acetone, 0·25 ml. |
| 7 November 1962* | 35–38 | Iodoacetic acid, 1·0 per cent (w/v) solution in acetone, 0·25 ml. |
| | 39–42 | Trichloroacetic acid, 5·0 per cent (w/v) solution in acetone, 0·25 ml. |
| | 43–46 | Mechanical abrasion, as described in text. |

* = Dates of injection with 25 mg. urethane.

### EXPERIMENTAL

Male mice were grouped at random into groups of twenty, with the provision for some mortality noted below in the case of the groups treated with cantharidin. The stage of the hair cycle was ignored.

*Experiment I*

The mice of groups 1 to 8 (Table I) were given no preliminary application or a preliminary application of acetone to the whole area of the skin of the back at 0, 12, 18, 24 hours, 2, 4 or 6 days before an injection of 25 mg. urethane, respectively. The animals of groups 9 to 12 were given an application of 0·25 ml. acetone to the right side of the skin of the back at 24 hours prior to the injection of 25 mg. urethane.

*Experiments II and III*

In random lots, the mice of six groups in Experiment II or of four groups in Experiment III, were given a preliminary treatment with one of the various irritants or scarification to the right side of the skin of the back at intervals of 1, 2, 3, 4, 5 or 6 days in Experiment II, or 1, 2, 3 or 5 days in Experiment III, respective to the groups, before the injection of 25 mg. urethane according to the schedule in Table I. Thus, for example, groups 13, 14, 15, 16, 17 and 18 were given a single application of 0·25 ml. of a 50 per cent solution of acetic acid in acetone to the right side of the skin of the back at intervals of 1, 2, 3, 4, 5 or 6 days respectively before the injection of urethane : and so on *mutatis mutandis* for each lot of six or four groups with each preliminary treatment.

In each experiment, from the seventh day after the injection of urethane the mice were painted over the whole area, that is both right and left sides, of the skin of the back with approximately 0·25 ml. of a 0·5 per cent solution of croton oil in acetone once each seventh day for twenty weeks.

At the time of each painting, or after the sixteenth week fortnightly, the numbers of survivors, the number of papillomata and their distribution on the skin of the back were recorded. The final count was made two weeks after the last application of croton oil.

The concentrations of the various irritants used for the preliminary treatment were based on the work of Berenblum (1935, 1941) concerning the influence of various chemicals when applied with repeated applications of carcinogenic hydrocarbons. Preliminary experiments were made to determine the effects of various concentrations on mouse skin and concentrations selected that resulted in considerable hyperplasia and scaling of the skin without gross ulceration. However, the mice treated with 50 per cent acetic acid were observed only for 6 days by which time ulceration had not occurred but, in the experiment proper, gross ulceration invariably occurred later. In the case of iodoacetic acid and cantharidin, higher concentrations also led to considerable mortality in the mice. Even with the concentration of cantharidin used, allowance had to be made for a 1 in 6 mortality to provide a full complement of twenty mice after one week, extra mice being disposed of at random before the first weekly application of croton oil.

Mechanical abrasion was carried out with the aid of the saw edge of a hacksaw blade held vertically to the skin with the long axis at right angles to the line of abrasion. With the clipped skin of the mice drawn taut, the blade was moved cranially and caudally along the area to be abraded until the superacial layers of skin were removed and the surface was moist and shiny due to serous oozing. The abrasion was usually not deep enough to produce bleeding. Alternative methods tested for abrasion using fine, medium or coarse glass paper were not as satisfactory because of the mobility of the skin.

### RESULTS

*Clinical effects of the irritants and scarification*

All the preliminary treatments produced acute inflammation in the treated area of skin. Oedema was apparent within 6 hours and developed most rapidly in the case of scarification, iodoacetic acid and cantharidin and least rapidly with turpentine. Twenty-four hours after the application the epidermis was less translucent probably because of epidermal proliferation since this appearance gradually gave place by the third or fourth day to a scaling hyperkeratosis that varied considerably in intensity between the different treatments. The oedema and epidermal changes were in all cases confined to the right side although often reaching the midline. No changes were observed on the left side of the skin of the back that had had no preliminary treatment.

The mice treated with cantharidin, iodoacetic acid or xylene in the amounts given showed some evidence of general toxic effects due to the drugs.

*Acetic acid.*—The treated skin was oedematous for about four days after application. By the third day the painted side appeared to be very tender to even light touch. A dry epithelial crust formed about the fourth day which separated from the sixth day onwards leaving an ulcerated surface that healed by the end of the third week. The extent of ulceration varied considerably but it was present in all mice at some time within the first two weeks. Healing of the ulcerated areas resulted in linear scars easily seen for the duration of the experiment as ridges of tissue in which short hairs persisted after clipping had removed the surrounding hair.

*Xylene.*—The oedema was easily observed in all the mice for four days. From the third day, dry scaliness became apparent and was present for about one week. The mice of these groups became very irritable after the application of xylene and engaged in a considerable amount of back-biting. No ulceration appeared to result from the xylene itself. The xylene appeared to have some toxicity for the mice, eight of which died in the two weeks after the application for no other cause.

*Vegetable turpentine.*—This produced the mildest response of all the irritants used. Oedema persisted for two to three days and scaling of the treated skin occurred from about the third to seventh day, but this was less than that produced by any of the other treatments.

*Cantharidin.*—All surviving mice showed severe oedema and tenderness for about five days in the painted area and the skin felt " leathery " between the palpating fingers. All the mice developed gross dry scaly hyperkeratosis from the third day. A few mice developed small areas of localized ulceration but there was no subsequent scarring. During the second week most mice lost hair in the area painted. The degree of epilation varied from complete baldness to only small hairless islands. Many remained hairless for four weeks and a few for longer. The mice of these groups also showed a generalized malaise, their eyes being a little sunken and their tails cold.

*Iodoacetic acid.*—Severe oedema and a curious pallor of the treated skin was apparent within 24 hours and was evident until the fourth day. Thereafter the affected skin remained thickened, firm and scaly for several days. At no stage did ulceration occur and by the end of two weeks the skin was similar to that on the untreated side. Three mice died from toxic effects of the drug within 24 hours of the application.

*Trichloroacetic acid.*—This produced considerable thickening and scaliness of the treated area of skin. There was no ulceration in the first seven days but during the second week a few small ulcers appeared on a few of the mice. This was not followed by scarring.

*Mechanical abrasion.*—Ulceration and oedema were subsiding and hair growth was apparent within two days of abrasion and the whole surface was re-epithelialized in most cases after six days. By two weeks the skin was healed but with some areas of flat pale scarring. This scarring was visible for the duration of the experiment but was not present in prominent ridges as was the scarring after acetic acid. During the first few days these mice consumed twice the normal amount of water.

From these macroscopic observations a subjective assessment of the relative severity of the local effects of the preliminary treatments was made before counting the tumour yields. In decreasing order acetic acid, mechanical abrasion, cantharidin and iodoacetic acid produced the most severe changes, trichloroacetic acid and xylene appeared to produce a similar less effect and turpentine produced the least severe changes. However the character of the sequence of changes varied with the different irritants, thus iodoacetic acid, cantharidin and xylene produced more oedema than trichloroacetic acid or acetic acid although the latter produced more ulceration especially in the later stages, so that a precise comparison of the effects was not possible. It is emphasised that this grading refers only to the effects actually produced by the various preliminary treatments in the concentrations employed. Acetone produced no obvious change in the skin at all.

It became obvious from as early as the tenth week of painting with croton oil that when tumours developed there was a preference for the side that had received the preliminary treatments in Experiments II and III, whereas in Experiment I no obvious influence of the prior application of acetone alone was apparent at any stage.

*Yield of Tumours*

The number of mice surviving after 22 weeks and the yield of tumours in the various groups are shown in Tables II and III.

Although considerable care was taken to ensure that the preliminary treatments were confined to the right side, the possibility that some spread might have

TABLE II.—*Influence of a Preliminary Application of Acetone before Injection of Urethane*

| Group | Interval between preliminary application and injection of urethane | Survivors | Mice with tumours: Number of mice | Number of tumours: Right side | Midline | Left side | Total |
|---|---|---|---|---|---|---|---|
| 1 | No prior application | 15 | 8 | 4 | 0 | 6 | 10 |
| 2 | 0 | 13 | 7 | 4 | 1 | 6 | 11 |
| 3 | 12 hours | 12 | 5 | 3 | 0 | 4 | 7 |
| 4 | 18 ,, | 15 | 6 | 5 | 0 | 4 | 9 |
| 5 | 24 ,, | 18 | 6 | 4 | 2 | 6 | 12 |
| 6 | 2 days | 17 | 4 | 5 | 2 | 3 | 10 |
| 7 | 4 ,, | 14 | 2 | 2 | 0 | 3 | 5 |
| 8 | 6 ,, | 19 | 6 | 5 | 1 | 4 | 10 |
| | Total | 123 | 44 | 32 | 6 | 36 | 74 |
| 9, 10, 11, 12 | 24 hours | 67 | 19 | 22 | 5 | 14 | 41 |

20 mice in each group at beginning of experiment.

occurred to the opposite side cannot be rigidly excluded. In fact, as noted above, changes in the skin following the preliminary treatments did appear to be confined to the right side. Even if this were so, any influence of the preliminary treatments would be manifest in the mid-line zones between the treated and untreated sides and also, in a proportion of the mice, some tumours would have appeared in this zone without prior stimulation so that the assignation of mid-line tumours is not possible. Since the purpose of the experiment was to compare an area of skin that had a preliminary treatment with an area that had no such treatment, it is reasonable and best to exclude the mid-line tumours from consideration and compare the two sides that represent similar areas differing only in the preliminary treatment.

Therefore, in Tables II and III, the data have been dissected, from the recorded distribution of the lesions, to show the number of tumours on the right side, on the left side and on the mid-line of the skin of the back. For this purpose the mid-line was considered as a band about 3 mm. wide separating the two sides.

It is clear from Table II that there is no significant variation in the number of tumours between the groups of mice given a preliminary application of acetone at various intervals before the injection of urethane ($\chi^2 = 3{\cdot}09$, 7 d.f., N.S.) nor between the number of tumours on each side of the skin of mice given a preliminary application of acetone to the right side 24 hours before injection of urethane ($\chi^2 = 1{\cdot}7$, 1 d.f., $0{\cdot}20 > P > 0{\cdot}10$), nor between the two sides of the mice in general.

In the case of the mice treated on the right side with the various irritants or scarification, Table III shows clearly that the number of tumours on the treated side is significantly greater than on the untreated side with each of the seven treatments. It is also evident that the number of mice with tumours on the treated side is greater than the number of mice with tumours on the untreated side, and that the number of tumours per mouse likewise is increased on the treated side. However, since these parameters are at least partly related to the number of tumours they are not considered in the further analysis.

The analysis is based on the number of tumours recorded on each side for each individual mouse. As the distribution of the number of tumours per mouse is probably Poisson, the square root transformation was used throughout. The score of each mouse was then the square root of the number of tumours on the left side or the right side as the case may be.

*Analysis of left sided tumours*

To test whether the unpainted sides had been affected by the treatments an analysis of the left side scores was made for the seven treatments in Experiments II and III and for days $-1$, $-2$, $-3$ and $-5$ giving a 7 by 4 factorial arrangement with unequal class numbers. There was a significant difference between the effects of the different treatments ($P < 0{\cdot}005$). The days effect was not significant nor was there any significant interaction between treatment and days.

The groups of mice treated with cantharidin, iodoacetic acid and xylene gave significantly higher yields than the groups treated with acetic acid, trichloroacetic acid, turpentine or scarification. Within the first group cantharidin gave a significantly higher yield than iodoacetic acid or xylene. These tests were performed at the 5 per cent level of significance.

TABLE III.—*Influence of Preliminary Treatment with Irritant Chemicals or Scarification before Injection of Urethrane*

| Preliminary treatment | Group | Interval between preliminary treatment and injection of urethane | Number of survivors | Mice with mid-line tumours: Number of mice | Mice with mid-line tumours: Number of tumours | Distribution of tumours per mouse on treated and untreated sides: Mice with no tumours | Distribution of tumours per mouse on treated and untreated sides: Number of tumours on right side / Number of tumours on left side |
|---|---|---|---|---|---|---|---|
| Acetic acid | 13 | −1 | 17 | 3 | 4 | 7 | 4/1 2/1 1/1 4/0 3/0 2/0 1/0 1/0 1/0 0/1 |
| | 14 | −2 | 15 | 0 | 0 | 5 | 3/1 2/1 4/0 4/0 3/0 2/0 1/0 1/0 1/0 0/1 |
| | 15 | −3 | 13 | 2 | 2 | 3 | 6/2 3/2 11/1 1/1 4/0 3/0 3/0 2/0 1/0 1/0 |
| | 16 | −4 | 17 | 0 | 0 | 7 | 1/2 6/0 4/0 3/0 2/0 2/0 1/0 1/0 1/0 0/1 |
| | 17 | −5 | 17 | 0 | 0 | 7 | 2/1 1/1 4/0 2/0 2/0 1/0 1/0 1/0 1/0 0/1 |
| | 18 | −6 | 17 | 1 | 2 | 10 | 1/2 1/0 1/0 1/0 1/0 0/1 0/1 |
| Xylene | 19 | −1 | 18 | 4 | 5 | 2 | 4/3 7/2 5/1 3/1 3/1 3/0 3/0 3/1 2/0 2/0 2/0 2/0 1/0 1/0 1/0 1/0 |
| | 20 | −2 | 12 | 4 | 4 | 1 | 6/3 1/3 4/2 4/1 3/1 10/0 4/0 4/0 1/0 1/0 1/0 |
| | 21 | −3 | 17 | 4 | 4 | 7 | 11/1 3/0 3/0 2/0 2/0 2/0 1/0 1/0 0/1 0/1 |
| | 22 | −4 | 17 | 4 | 6 | 8 | 1/2 3/1 2/1 3/0 3/0 3/0 2/0 1/0 1/0 |
| | 23 | −5 | 14 | 0 | 0 | 5 | 8/5 3/2 3/1 5/0 3/0 2/0 1/0 0/1 0/1 |
| | 24 | −6 | 15 | 1 | 1 | 6 | 2/0 2/0 2/0 1/0 1/0 1/0 1/0 1/0 1/0 |
| Turpentine | 25 | −1 | 17 | 1 | 1 | 10 | 5/1 5/1 3/1 2/1 1/1 4/0 1/0 |
| | 26 | −2 | 18 | 1 | 1 | 5 | 4/2 3/1 1/1 4/0 3/0 3/0 2/0 2/0 1/0 1/0 1/0 1/0 1/0 |
| | 27 | −3 | 19 | 1 | 1 | 9 | 5/0 4/0 4/0 3/0 1/0 1/0 1/0 0/1 0/1 0/1 |
| | 28 | −4 | 19 | 2 | 2 | 10 | 3/2 1/1 2/0 2/0 1/0 1/0 1/0 0/2 0/1 |
| | 29 | −5 | 19 | 1 | 1 | 12 | 3/0 2/0 1/0 1/0 1/0 0/1 0/1 |
| | 30 | −6 | 18 | 0 | 0 | 12 | 1/1 2/0 1/0 1/0 0/1 0/1 |
| Cantharidin | 31 | −1 | 16 | 5 | 5 | 4 | 4/4 2/2 9/1 6/1 3/1 2/1 1/1 2/0 2/0 1/0 1/0 0/1 |
| | 32 | −2 | 20 | 3 | 3 | 6 | 10/3 4/3 4/3 11/1 2/1 1/1 8/0 8/0 7/0 6/0 3/0 3/0 2/0 1/0 |
| | 33 | −3 | 19 | 9 | 11 | 3 | 9/3 3/3 7/2 4/2 3/2 2/2 2/2 6/1 6/1 5/1 5/0 4/0 4/0 2/0 1/0 0/1 |
| | 34 | −5 | 20 | 3 | 5 | 7 | 10/2 4/2 2/1 2/1 3/0 3/0 2/0 2/0 2/0 2/0 1/0 1/0 0/1 |
| Iodoacetic acid | 35 | −1 | 19 | 3 | 3 | 7 | 11/2 4/2 11/1 5/1 3/1 6/0 5/0 2/0 1/0 1/0 1/0 1/0 |
| | 36 | −2 | 19 | 3 | 3 | 5 | 8/2 7/2 2/2 1/2 9/1 7/1 4/1 1/1 2/0 2/0 2/0 1/0 0/1 0/1 |
| | 37 | −3 | 17 | 2 | 3 | 4 | 9/2 5/2 4/2 6/1 3/1 2/1 8/0 6/0 3/0 2/0 2/0 1/0 1/0 |
| | 38 | −5 | 18 | 1 | 2 | 6 | 3/3 1/2 5/1 3/1 3/1 3/1 1/1 2/0 1/0 1/0 1/0 1/0 |
| Trichloroacetic Acid | 39 | −1 | 18 | 1 | 2 | 11 | 2/0 2/0 2/0 1/0 1/0 1/0 1/0 |
| | 40 | −2 | 19 | 2 | 2 | 3 | 6/2 6/2 4/1 2/1 2/1 1/1 2/0 2/0 2/0 2/0 2/0 1/0 1/0 1/0 0/2 0/1 |
| | 41 | −3 | 18 | 4 | 4 | 8 | 4/1 1/1 6/0 4/0 3/0 3/0 3/0 1/0 1/0 1/0 |
| | 42 | −5 | 16 | 1 | 1 | 6 | 3/1 2/0 1/0 1/0 1/0 1/0 0/1 0/1 0/1 0/1 |
| Scarification | 43 | −1 | 16 | 2 | 2 | 3 | 4/1 2/1 1/1 3/0 3/0 3/0 3/0 3/0 2/0 2/0 1/0 1/0 0/1 |
| | 44 | −2 | 17 | 2 | 2 | 3 | 3/2 2/2 6/1 3/1 2/1 1/1 7/0 4/0 3/0 3/0 3/0 2/0 2/0 2/0 |
| | 45 | −3 | 15 | 1 | 1 | 5 | 4/1 1/1 4/0 3/0 2/0 1/0 1/0 1/0 1/0 1/0 |
| | 46 | −5 | 17 | 2 | 2 | 10 | 5/1 2/1 3/0 1/0 0/1 0/1 0/1 |

*Analysis of right-sided tumours*

The yield of tumours on the right side showed a significant correlation with the yield on the left side. To examine the way in which the treatments produced their effects an analysis of covariance was undertaken for the 3 by 6 factorial involving acetic acid, xylene and turpentine, Experiment II, and a similar analysis for the 4 by 4 factorial involving cantharidin, iodoacetic acid, trichloroacetic acid and scarification, Experiment III.

In the first analysis it was found that after the correlation with the left sides was eliminated, the treatment effect was still significant ($P < 0{\cdot}005$). There were significant differences between days ($P < 0{\cdot}005$) but there was no interaction between days and treatments. The treatment means were adjusted for regression on the left-hand side yield and these adjusted means were ranked in descending order as xylene, acetic acid and turpentine. The differences between each pair were found to be significant. The maximum adjusted mean for the day's effect occurred for day $-2$.

For the second set of treatments, Experiment III, the covariance analysis again showed a significant treatment effect ($P < 0{\cdot}05$), a significant day's effect ($P < 0{\cdot}005$) and no significant interaction between days and treatments. The adjusted treatment means were ranked in descending order as cantharidin, iodoacetic acid, scarification and trichloroacetic acid. The appropriate tests then showed that the adjusted yields for cantharidin and iodoacetic acid were significantly greater than for trichloroacetic acid, no other treatment differences being significant. The maximum day effect was for day $-3$ followed closely by day $-2$.

Care must be taken in interpreting analyses of covariance when the control variable (that is the tumour yield on the side that had no preliminary treatment) has apparently been effected by the treatments. If it is assumed that there was a systemic effect which was reflected in the variation of the left side tumours from treatment to treatment, this effect is presumably removed by the covariance analysis. The significant variability that remains among the adjusted treatment means indicates that the postulated systemic effect does not measure the whole of the relative effects of the different treatments but that the direct effects on the painted sides still vary from treatment to treatment in the manner described.

*Time of appearance of tumours*

The mean of the times of appearance of each tumour on the untreated side was 16·4 weeks over all the mice in all the groups and varied from 13 weeks to 20 weeks for the mice of the individual groups, whereas on the treated side it was 14·5 weeks over all the groups and varied from 12 weeks to 19 weeks between the groups. However the mean times of appearance of the tumours would appear to to partly related to the other parameters and since it is doubtful if differences of this order would be of biological significance in experiments of this design, no statistical analysis has been undertaken. As would be expected, the first tumours to appear occurred earlier on the treated side but this was not invariably the case.

*Distributions of papillomata*

In general the papillomata appeared to be randomly distributed over the skin of the different areas considered. However, in the case of groups 13 to 18

in which the mice were treated with acetic acid on the right side, there was an obvious concentration of papillomata along the margins of the prominent linear scars, 37 of the 116 tumours on the treated side appearing in this situation. There was no demonstrable relationship between the site of development of tumours and the pale flat scars on the right side of the skin of the back of the mice subjected to mechanical abrasion (groups 43 to 46).

### DISCUSSION

In the first place, Experiment I shows that a single application of acetone to the skin of mice at various intervals preceding the injection of urethane has no significant influence on the tumour yield after subsequent promoting treatment with croton oil. The augmenting influence of a solution of croton oil in acetone (Pound, 1962 ; Pound and Bell, 1962 ; Pound, 1963) is therefore to be ascribed firmly to the croton oil. In this respect it is significant that acetone produced no clinical evidence of inflammation or cellular proliferation in the skin and that Pound (1962) found no significant histological change in the skin of mice that had been given twenty weekly applications of acetone. As a corollary, in view of the results with various irritants, it follows that even a small amount of a highly irritant impurity in the acetone might, in certain circumstances, lead to an augmented tumour yield.

Secondly, in contrast to the lack of any influence of the acetone solvent, Experiments II and III show that the yield of tumours was increased by preliminary treatment of the skin with acetic acid, xylene or turpentine in Experiment II, or with cantharidin, iodoacetic acid, trichloroacetic acid or scarification in Experiment III, on one side of the skin of the back at various intervals before the injection of urethane. The tumour yield on the treated side, as compared to the untreated side, was significantly augmented when the urethane was injected 24 hours later, reached a maximum at an interval of two or three days and was declining by the fifth or sixth day. The overall pattern of the effect therefore appears to be similar to the augmenting influence of a preliminary application of croton oil reported by Pound and Bell (1962) and Pound (1963).

In the present work the changes in the skin produced by the irritants, as observed macroscopically, were graded in descending order of severity : (*a*) acetic acid, mechanical abrasion, cantharidin and iodoacetic acid ; (*b*) trichloroacetic acid and xylene ; (*c*) turpentine. This grading appears to agree tolerably with the ranking of the maximum augmenting effects on the tumour yields, namely xylene, acetic acid and turpentine in Experiment II and cantharidin, iodoacetic acid, scarification and trichloroacetic acid in Experiment III. The augmenting influence of a 0·5 per cent solution of croton oil in acetone was insignificant by the third day (Pound and Bell, 1962). If a larger dose of croton oil (0·7 per cent in acetone) is applied, the skin changes are more severe and comparable with the effect of the irritants used in the present work and the augmenting influence then persists for about five days (Pound, unpublished data).

The above features all point to the conclusion that the local augmenting effect is associated with the local phenomena produced by the " irritants " and is related to the severity of them irrespective of the means used which probably act by different biochemical mechanisms. More generally, it appears likely that any means that produce these phenomena at an appropriate time would augment the tumour yield. The common feature of the effective preliminary treatments is to produce inflammation almost at once and after a delay of some hours cellular proliferation leading to scaling hyperkeratosis in the skin over the next four or five days.

Increased availability of urethane to the skin, consequent upon vascular dilatation, does not appear to be the significant factor. Thus the urethane was injected subcutaneously between the scapulae and might be expected to produce a local concentration in the skin of the fore half of the back and so increase the tumour yield in this area. However the tumours were randomly distributed over the fore and rear halves of the back as was found similarly by Pound (1963). The augmenting effect is therefore probably bound up with cellular proliferation in the skin, with the implication that dividing cells may be susceptible to the initiating action of urethane.

The irritants used in this work were selected because, in addition to the production of inflammation and cellular proliferation in the skin by different mechanisms, other relevant properties of them were known. Trichloroacetic acid, acetic acid, iodoacetic acid and turpentine did not influence the tumour yield when applied to mouse skin alternately with tar (Berenblum, 1935). Turpentine and xylene failed to influence the tumour yield when applied together with or alternately with benzopyrene and were not carcinogenic when applied alone (Berenblum, 1941). Iodoacetic acid was found to be a mild promoting agent after initiating treatment of mouse skin with 9,10-dimethyl-1,2-benzanthracene while acetic acid, turpentine and cantharidin had no promoting effect and are presumably not carcinogenic (Gwynn and Salaman, 1953). However, in the case of initiation by urethane, the possibility that materials other than croton oil might exert promoting activity does not appear to have been investigated. With this limitation it is clear from the present experiments that the augmenting effect is not related to the promoting capacity of any material nor to any weak carcinogenic property as might be suggested in the case of croton oil. Cantharidin had an anti-carcinogenic effect when applied alternately with repeated applications of tar (Berenblum, 1935) but no such property is manifest in the present experiment in which the converse effect was found on initiation by urethane.

It is of interest that the local application of the irritants led to variation of the tumour yields on the untreated side. The yields on the untreated sides were increased by cantharidin, iodoacetic acid and xylene in descending order. It must be assumed that random variations and genetic variations in the susceptibility of the mice to develop tumours in this experimental system are eliminated by random selection of the mice into groups, although the latter would be one source of an overall correlation between the tumour yields on the two sides in individual mice within the groups. The second factor to produce this influence in the untreated side would be spread of the preliminary treatment to the untreated side. Reasons have been advanced above for believing that this did not occur. Further evidence against this as a cause of the variation is the fact that there was no significant variation between days as on the sides locally treated. Thirdly, the remaining explanation is that cantharidin, iodoacetic acid or xylene exerted a systemic effect that influenced the tumour yield and it is relevant that these substances were those that showed evidence of toxicity to the mice. It is clear that the source of the variation of the tumours on the untreated sides is a matter for further investigation.

Lastly the concentration of tumours along the lines of scarring produced by acetic acid should receive some comment. This result is best interpreted only as a concentration of tumours in an area where ulceration has been severe with resulting increased proliferation in the tissues.

### SUMMARY

1. Mice were injected with urethane as a tumour initiator and subsequently painted once each week for twenty weeks with croton oil over the whole area of the skin of the back as a promoting treatment.

2. Groups of these mice were either scarified, or given a single preliminary application of acetic acid, trichloroacetic acid, iodoacetic acid, xylene, turpentine or cantharidin on the right side of the skin of the back at various intervals before the injection of urethane. The preliminary treatments, as judged by the epidermal hyperplasia and inflammation produced, were confined to the treated area. Other groups of mice were treated with acetone to the skin of the back before the administration of the urethane, the acetone produced no gross change in the skin.

3. Prior application of acetone did not influence the tumour yield.

4. The yield of tumours in the mice treated by scarification or the various chemicals was greater on the treated side than on the untreated side. The augmenting influence was significant if the interval between the preliminary application and injection of urethane was 24 hours, reach a maximum at an interval of two or three days and declined by the fifth or sixth day.

5. The local augmenting effect of the several treatments appears to be associated with, and related to the severity of, the local phenomena produced in the skin.

6. In addition to the local augmenting influence, preliminary treatment of the skin with cantharidin, iodoacetic acid or xylene appeared to have a general effect that influenced the tumour yields.

The senior author (A.W.P.) wishes to thank Professor I. Berenblum, Department of Experimental Biology, The Weizmann Institute of Science, Rehovoth, Israel, for the generous gift of a sample of croton oil that made this work possible ; and Dr. H. Silverstone, Reader in Medical Statistics, Department of Social and Preventive Medicine, the University of Queensland, for the statistical analysis.

### REFERENCES

BERENBLUM, I.—(1935) *J. Path. Bact.*, **40**, 549.—(1941) *Cancer Res.*, **1**, 44.
BOUTWELL, R. K., BOSCH, DOROTHY AND RUSCH, H. P.—(1957) *Ibid.*, **17**, 71.
GWYNN, R. H. AND SALAMAN, M. H.—(1953) *Brit. J. Cancer*, **7**, 482.
POUND, A. W.—(1962) *Ibid.*, **16**, 246.—(1963) *Aust. J. exp. Biol. med. Sci.*, **41**, 73.
*Idem* AND BELL, J. R.—(1962) *Brit. J. Cancer*, **16**, 690.
ROE, F. J. C.—(1956) *Ibid.*, **10**, 72.
*Idem* AND PEIRCE, WINIFRED E. H.—(1961) *Cancer Res.*, **21**, 338.
SALAMAN, M. H.—(1958) *Brit. med. Bull.*, **14**, 116.